# Heat Stress Is More Damaging to Superior Spikelets than Inferiors of Rice (*Oryza sativa* L.) due to Their Different Organ Temperatures

**DOI:** 10.3389/fpls.2016.01637

**Published:** 2016-11-08

**Authors:** Guanfu Fu, Baohua Feng, Caixia Zhang, Yongjie Yang, Xueqin Yang, Tingting Chen, Xia Zhao, Xiufu Zhang, Qianyu Jin, Longxing Tao

**Affiliations:** State Key Laboratory of Rice Biology, China National Rice Research InstituteHangzhou, China

**Keywords:** heat injury, inferior spikelets, organ temperatures, rice (*Oryza Sativa* L.), superior spikelets

## Abstract

In general, the fertility and kernel weight of inferior spikelets of rice (*Oryza Sativa* L.) are obviously lower than those of superior spikelets, especially under abiotic stress. However, different responses to heat stress are seemed to show between the superior and inferior spikelet, and this response is scarcely documented that the intrinsic factors remain elusive. In order to reveal the mechanism underlying, two rice plants with different heat tolerance were subjected to heat stress of 40°C at anthesis. The results indicated that a greater decrease in fertility and kernel weight was observed in superior spikelets compared to inferior spikelets. This decrease was primarily ascribed to their different organ temperatures, in which the temperature of the superior spikelets was significantly higher than that of inferior spikelets. We inferred the differences in canopy temperature, light intensity and panicle types, were the primary reasons for the temperature difference between superior and inferior spikelets. Under heat stress, the fertility and kernel weight of superior and inferior spikelets decreased as the panicle numbers per plant were reduced, which was accompanied by significantly increasing the canopy temperatures. Thus, it was suggested that the rice plant with characteristic features of an upright growth habit and loose panicles might be more susceptible to heat stress resulting from their higher canopy and spikelets temperatures.

## Introduction

Spikelets grown within the same panicle of rice (*Oryza Sativa* L.) can be classified as superior spikelets and inferior spikelets, in which the former are located on apical primary branches, while the latter are located on proximal secondary branches (Mohapatra et al., 1993; Ishimaru et al., 2003). The superior spikelets flower earlier, fill faster and produce larger and heavier grains. In contrast, the inferior spikelets flower later, exhibit a slower rate of increase in dry weight during grain development and a lower grain weight than superior spikelets (Yang et al., 2006; Dong et al., 2014). Furthermore, poor fertility has been shown in the inferior spikelets (Wang et al., 2001, 2003; Cheng et al., 2003; Tao et al., 2003). Unfortunately, the differences in spikelet fertility, kernel weight and grain quality between the superior and inferior spikelets are more exacerbated in large panicle rice, especially the super rice (Mohapatra et al., 2009; Zhang et al., 2009). These super cultivars often fail to achieve the high yield mainly ascribed to the poor grain-filling of later-flowering inferior spikelets (Peng et al., 1999; Yang et al., 2002).

Many factors, such as assimilation supply, hormonal balance (Yang et al., 2006; Zhang et al., 2009; Yang and Zhang, 2010), and activities and gene expressions of key enzymes involved in sucrose-to-starch conversion (Jeng et al., 2003; Ishimaru et al., 2005; Kato et al., 2007; Wang et al., 2012), are involved in mediating the grain-filling of inferior spikelets. Most studies have shown that the low activities of key enzymes in carbon metabolism rather than carbon limitation mainly contribute to the poor grain-filling of inferiors because their enzyme activities are markedly lower (Yang J.C. et al., 2001; Yang et al., 2002; Tao et al., 2003), whereas the poor fertility in inferior spikelets may be associated with a lack of assimilates (Wang et al., 2001; Tao et al., 2003; Kobata et al., 2013). It was found that spikelet fertility could be easily decreased under adverse conditions of assimilation around flowering, while halving the density or removing the upper panicle could significantly increase the fertility in inferior spikelets (Kobata et al., 2013). Within a panicle, the superior spikelets can inhibit the inferiors, and this apical spikelets superiority of superior spikelets over inferior spikelets can partly be demonstrated by application of the exogenous auxin (IAA) (Wang et al., 2001, 2003). Furthermore, different expressions and functions of miRNAs exist between the superior and inferior spikelets (Peng et al., 2011, 2014).The interaction among the factors inside spikelets, including the hormones, mRNA transcriptions, protein expressions, and activities of key enzymes involved in sucrose-to-starch conversion, regulate the grain-filling process of superior and inferior spikelets (Yang et al., 2006; Zhang et al., 2009; Yang and Zhang, 2010). Additionally, poorly developed vascular bundles linked to inferior spikelets may be another important factor resulting in slow or aborted grain-filling because it can obviously decrease the total RNA and mRNA in the inferior spikelets ovaries (Huang et al., 2005).

For decades, the inferior spikelets of rice were reported to be more vulnerable to abiotic adversities, including drought stress (Zhang et al., 2012), heat damage (Dong et al., 2011), and cold injury (Yuan et al., 2006). When subjected to severe drought stress, no significant decrease was observed in the endosperm cell division rate, seed-setting rate, grain-filling rate, and grain weight in the earlier flowering superior spikelets, whereas those in the later flowering inferior spikelets were significantly reduced (Chen et al., 2013). This finding agreed with the results of Tao et al. (2010) and Yang W.B. et al. (2014), in which the former reported that higher increases in spikelet sterility were shown in the inferior spikelets compared to the superiors and the latter indicated that decreases in the kernel weight of inferior spikelets was higher than that of superiors when subjected to water deficit. Effects of low temperature at an early grain-filling stage on the grain shape, structure of endosperm and the chalkiness area of the inferior spikelets were serious, whereas those of superior spikelets were not obvious (Yuan et al., 2006). Nevertheless, the results of Dong et al. (2011) indicated that the influence of high temperature on grain quality varied with spikelet positions. When a high temperature was imposed at the initial grain-filling stage of rice, the spikelets on the primary branches showed a larger effect than those on the secondary branches, while in the same branch, more damages were observed to the later-flowering spikelets than the early-flowering spikelets. This finding means that the damages to the superior and inferior spikelets caused by heat stress may be different from those under other abiotic stresses. However, a major problem is that the response of superior and inferior spikelets to heat stress is scarcely documented and the intrinsic factors remain elusive.

Heat stress is defined as the rise in temperature beyond a critical threshold for a period of time that is sufficient to cause irreversible damage to plant growth and development (Wahid et al., 2007). This stress can dramatically reduce agricultural products, resulting in widespread risk of food insecurity and social problems. The previous results have indicated that expression of heat shock proteins (HSPs) can improve the tolerance of transgenic plants to heat shock (Qu et al., 2013; Mishra and Grover, 2015). Recently, two important genes, including *OgTT1* and *ERECTA* which play important role in thermo-tolerance, were identified in rice, *Arabidopsis*, and tomato (Li et al., 2015; Shen et al., 2015). These proteins or genes involved in conferring thermo-tolerance may be the approaches the plants evolved to survive in heat stress. Unfortunately, most of those proteins or genes are identified from rice seedlings or at the vegetative stage, and very few are found at the reproductive phase, particularly at anthesis. These findings suggest more complexity of heat tolerance in the flowering stage of rice. It is well-known that the flowering stage of rice is most sensitive to heat stress (Satake and Yoshida, 1978), any one of the factors, including the decrease in pollen viability and pollen germination, abnormal anther dehiscence, lack of pollen reaching the chapter, and impaired pollination induced by heat stress, can lead to floret sterility and yield loss (Matsui and Omasa, 2002; Matsui et al., 2005; Jagadish et al., 2010). Fortunately, rice plants can escape from heat stress by reducing the canopy temperature. This is another important way that rice plants evolve to reduce the damage caused by heat stress whether it is active or passive.

The canopy temperature measurement has been used to evaluate crop tolerance under drought stress and high temperature conditions (Rashid et al., 1999; Siebert et al., 2014; Pinto and Reynolds, 2015). It has been proposed that the organ temperature should be used to describe thermal damage rather than air temperature (Sheehy et al., 1998) because air temperature is a poor guide to the temperature of an object, especially the plant organs. Rice plant temperatures are not only depending on the organ position, size, shape, surface area, and hull morphology but also influenced by the air temperature, air humidity, radiation, and plant type (Sheehy et al., 1998; Yan et al., 2008, 2010). The results of Zhang et al. (2016) indicated that moderate heat stress of 40°C significantly decreased the spikelet fertility, whereas it caused little damage to the photosynthesis in flag leaves at anthesis because when the panicle temperature was above 38°C, the flag leaves were only approximately 34°C. This indicated that the damage degree induced by heat stress to rice plant organs mainly varied with their different temperatures. Therefore, the temperature difference may be the main factor responsible for the different responses of superior and inferior spikelets to heat stress. We have observed that a greater decrease in fertility and kernel weight were shown in the superior spikelets than inferior spikelets when heat stress occurred at anthesis, which suggested that heat stress caused more damage to the superior spikelets than inferior spikelets. This finding was very interesting because it was obviously different from previous results (Yuan et al., 2006; Dong et al., 2011; Zhang et al., 2012; Yang D.Q. et al., 2014; Zhao et al., 2014). Thus in this study, the spikelets and canopy temperatures, light intensity, relative humidity, spikelet transpiration rate, and other related physiology indexes were analyzed to reveal the intrinsic factors contributing to the more damage to the superior spikelet than inferior spikelets caused by heat stress.

## Materials and Methods

### Plant Materials and Heat Stress Treatments

**FIGURE 1:**
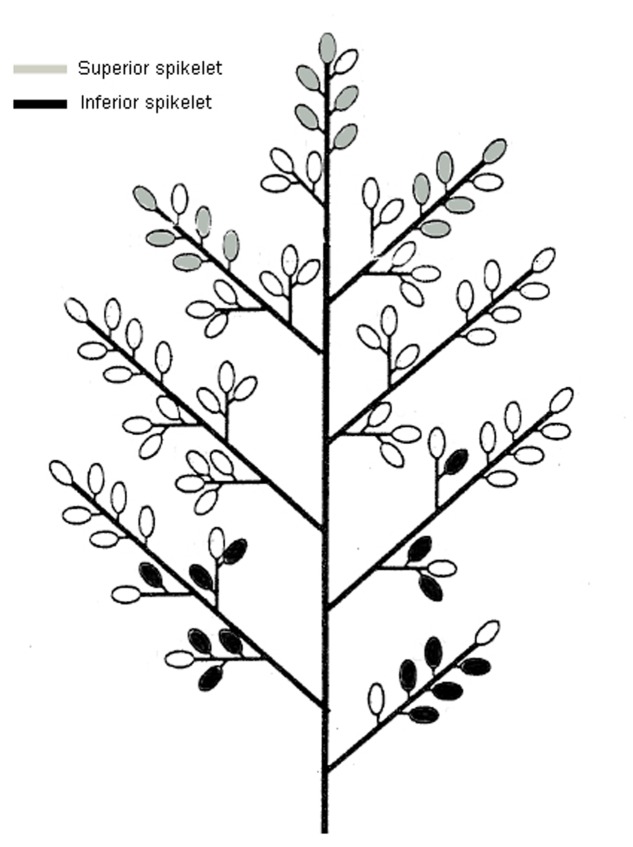
**Diagrams of the spikelets on the rice panicle (Kikuya et al., 2001 with some modifications).** The spikelets shown in gray represent the location of superiors and those shown in black represent the location of inferior spikelets.

Pot experiments (30 cm in diameter and 30 cm in height) were carried out at the Fuyang farm of the China National Rice Research Institute, Hangzhou, Zhejiang Province, China, from March to October in 2014. Each pot was filled with 15 kg of paddy soil blended with 20 g of compound fertilizer (N:P:K = 15:10:14). Two rice genotypes differing in heat resistance, i.e., V20 (heat resistance) and Zhong9 (heat susceptible), were directly sown in pots with eight seedlings per pot after germination. The seedlings at the 5.0–5.5 leaf stage were thinned to three leaves and grown outdoors until heat stress was imposed. The rice plants were subjected to heat stress of 40°C for 7 h from 9:30 to 15:30 every day from anthesis for 5, 10, and 15 days. The temperature was monitored and regulated by an automatic temperature control system with night and day temperatures of 30 and 40°C under heat stress, while the temperature was set at 24 and 33°C (night/day, respectively) in another greenhouse as controls. Both heat stress plants and the controls were exposed to normal sunlight. Six panicles (SP) were kept on each rice plant before heat stress was imposed, and about 1–3 small tillers were removed in each rice plant. However, to reveal the effect of the canopy temperature on the fertility and kernel weight in the superior and inferior spikelets, another two plant densities were subjected to heat stress for 10 days via controlling the panicles for each rice plant, including four panicles per plant (FP), and two panicles per plant (TP). In these two treatments, the main panicle and bigger panicles were kept, and the others were removed before heat stress was imposed. During the temperature treatment, the organ and canopy temperature, pollen viability, pollen numbers and pollen germinating on the stigma, reactive oxygen species (ROS), soluble sugars, starch branching enzyme activity, and phytohormones of superior and inferior spikelets were determined. Superior and inferior spikelets were sampled according to the methods of Peng et al. (2011), as described in **Figure 1**.

### Thermal Imaging of Superior and Inferior Spikelets

The temperatures of superior and inferior spikelets of rice with or without flag leaves were determined from 9:30 am to 10:30 am using an FLIR ThermaCAM^TM^ S65 system (FLIR Systems Inc., Portland, OR, USA) with a wide-angle camera lens (18 mm IR-LENS). The spectral range of this camera was 7.5–13 mm, and the detectable temperature range was –40 to 1,500°C. Temperature differences of less than 0.06°C (30°C, 50 Hz) could be detected with this camera. The camera was set up 0.5 m away from the rice plants. A black cloth was used to minimize the interference from other substances when recording the temperature of superior and inferior spikelets. The data were analyzed with Therma CAM Researcher Pro 2.7 software (FLIR Systems Inc., Portland, OR, USA).

### The Air Temperature, Relative Humidity, and Illumination within the Canopy

A meteorological automatic recorder (ZDR-34, Hangzhou, China) was placed in the middle of the rice plant populations to record the canopy temperature, relative humidity, and illumination intensity. For each rice genotype, two different vertical heights based on the canopy height in which superior and inferior spikelets were located were established. The temperature, relative humidity, and illumination intensity were recorded every 3 min.

### Relative Water Content and Transpiration Rate of Spikelet

The superior spikelet and inferior spikelet were collected on day 2–4 of heat stress treatment, and their relative water content (RWC) were determined according to the equation RWC = 100(FW – DW)/(TW – DW), where FW was fresh weight, DW was dry weight and TW was turgid weight. Turgid weight was determined after immersing the sample in the distilled water for 24 h at 48°C in darkness; dry weight was measured after oven-drying the sample for 48 h at 85°C.

The transpiration rate of the superior and inferior spikelet were analyzed at 10:00 am to 11:00 am on day 2–4 of the heat stress treatment with a Li-Cor 6400 portable photosynthesis system (Li-Cor Inc., Lincoln, NE, USA) under the following conditions: photosynthetic photon flux density of 1200 μmol m^–2^s^–1^; ambient CO_2_ (450 μmol mol^–1^); 500 μmol s^–1^ flow speed; and temperatures according to the treatment. The spikelets were kept in the leaf chamber of the instrument for 10 min. The spikelet areas were estimated by SC-G rice grain appearance quality image analysis system developed by Hangzhou WSeen Detection Technology Co., Ltd, China (Yin et al., 2015).

### Pollen Viability

Pollen viability was determined using the method of Gunawardena et al. (2003). Mature pollen grains collected from the spikelets before flowering at 4 days after heat stress were stained with potassium iodide/iodine solution (KI/I_2_). Pollen grains were removed from the anthers of the florets, placed into a drop of KI/I_2_ on a glass slide, and observed and photographed under a light microscope (DM4000B, Leica, Wetzlar, Germany).

### Pollen Numbers and Pollen Germinating on Stigma

More than 50 flowering spikelets were collected at 10:00–11:00 am on day 4 of the heat stress treatment. The pollinated stigmas were fixed in Carnoy’s fixing reagent (by volume: 30% chloroform, 10% acetic acid, and 57% ethanol). The samples were then washed with water, incubated in 10 mol L^–1^NaOH for 6–10 min at 56°C, and then stained in 0.1% (w/v) aniline blue solution for 24 h. The pollen numbers and pollen germinating on the stigma were observed and photographed at 350 nm with a fluorescence microscope (DM4000B, Leica, Wetzlar, Germany).

### ROS

For visualization and analysis of stigma ROS, the oxidation-sensitive probe DCFH-DA was used, as previously described by Sanchez et al. (1997). The pollinated stigmas were collected from the flowering spikelets and were immediately incubated with 5 M DCFH-DA. The fluorescence intensity was measured after 30 min incubation with 5 M DCFH-D by a fluorescence microscope (DM4000B, Leica, Wetzlar, Germany).

### Soluble Sugar, Starch Branching Enzyme Activity, and Phytohormones

The superior and inferior spikelets were collected on day 14 after heat stress to determine the soluble sugar concentrations, starch branching enzyme activities, and phytohormones. The concentration of soluble sugar was measured using the phenolsulfuric acid method (DuBois et al., 1956). Frozen superior and inferior grains (0.5 g) collected on day 14 after heat stress were homogenized in deionized water, and the extract was filtered and treated with 5% phenol and 98% sulfuric acid. The mixture was incubated for 1 h and absorbance at 485 nm was determined. The determination of starch branching enzyme (Q enzyme) activity was based on the methods of Zhao F.M. et al. (2007) and Zhao H.Y. et al. (2007). The methods and ELISA kit for determining the concentration of phytohormones, including IAA, ABA, GA_3,_ and ZR (zeatin riboside), were provided by Yang Y.M. et al. (2001) from the China Agricultural University, Beijing, China.

### Spikelet Fertility and Kernel Weight

At maturity, heat stressed and control rice plants were harvested, dried at 50°C for 48 h, and then the fertility and kernel weight of superior and inferior spikelets were determined.

### Statistical Analyses

Data were processed with SPSS 11.5 for Windows, and each value of mean and standard error in the figures represent four replications (unless otherwise stated). Tukey’s least significant difference (LSD) at a probability level of 5% was used to compare the differences between treatments and genotypes.

## Results

### Spikelet Fertility and Kernel Weight

**FIGURE 2:**
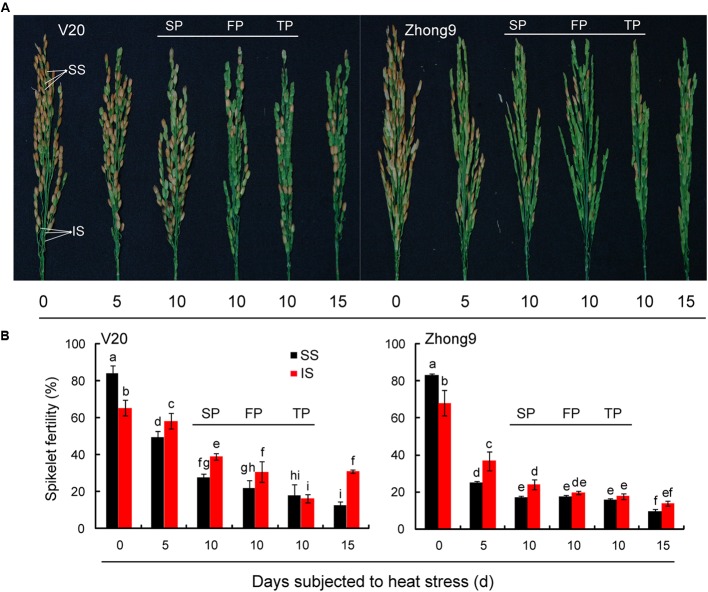
**Effect of heat stress on the spikelets fertility of superior and inferior spikelets of rice at anthesis.** These rice plants were subjected to heat stress of 40°C for 5, 10, and15 days, respectively, from anthesis, and determined at maturity. **(A)** The pictures of panicles with superior and inferior spikelets in both genotypes under control condition and heat stress condition. **(B)** The mean data of fertility of superior and inferior spikelets in both genotypes under control and heat stress. SS and IS were indicated as superior spikelets and inferior spikelets, respectively. 0 day was indicated as control that rice plants without heat stress imposed. Six panicles (SP) were controlled in each rice plants except for the treatments of FP and TP. SP, FP, and TP present each rice plant possessing six panicles, four panicles, and two panicles, respectively. Vertical bars denote standard deviations (*n* = 3). Different letters indicate significant differences between the superior spikelet and inferior spikelets in the same cultivar (*P* < 0.05).

Undoubtedly, the superior spikelets of rice exhibited greater fertility than inferiors at normal temperature, however, the results were reversed under heat stress at anthesis (**Figure 2**). The fertility of inferior spikelets were significantly higher than those of superiors in both rice genotypes when the rice plants were subjected to heat stress from anthesis for 5, 10, and 15 days. It is worth noting that a remarkable reduction in spikelet fertility was attained as the panicle numbers were reduced under heat stress for 10 days, especially for the inferior spikelets. The highest fertility of inferior spikelets was observed in the rice plants with SP; followed by those with FP (four panicles) and TP (two panicles).

**FIGURE 3:**
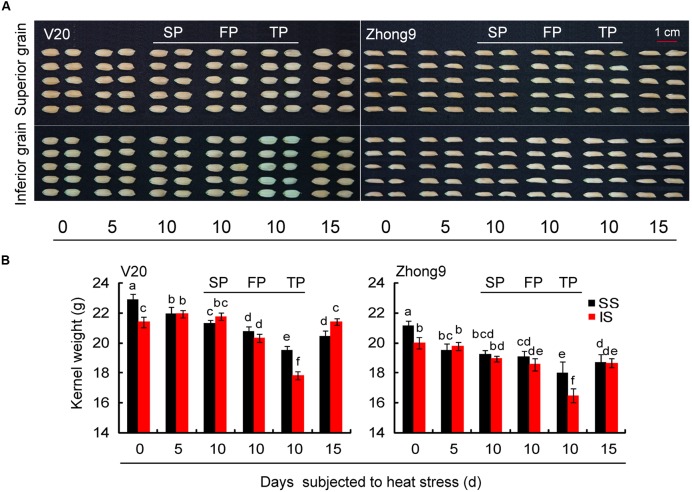
**Effect of heat stress on the kernel weight of superior and inferior spikelets of rice at anthesis.** These rice plants were subjected to heat stress of 40°C for 5, 10, and 15 days after flowering, and determined at maturity. **(A)** The pictures of the superior and inferior grains in both genotypes under control and heat stress. **(B)** The mean data of kernel weight of superior and inferior grains in both genotypes under control and heat stress. SS and IS were indicated as superior spikelets and inferior spikelets, respectively. 0 day was indicated as control that rice plants without heats stress imposed. SP were controlled in each rice plants except for the treatments of FP and TP. SP, FP, and TP present each rice plant possessing six panicles, four panicles, and two panicles, respectively. Vertical bars denote standard deviations (*n* = 3). Different letters indicate significant differences between the superior spikelets and inferior spikelets in the same cultivar (*P* < 0.05).

Similar to the changed pattern of spikelet fertility, the kernel weight of superior spikelets was also significantly higher than that of inferior spikelets in both control group genotypes (**Figure 3**). Nevertheless, for those rice plants subjected to heat stress for 5, 10, and 15 days, a greater reduction was also observed in the superior spikelets than inferiors compared with their respective controls. This effect diminished as the panicle numbers per plant decreased, especially for inferior spikelets. Compared with controls, higher decreases were observed in those rice plants with FP or TP than those with SP under heat stress.

### Spikelets Temperature

**FIGURE 4:**
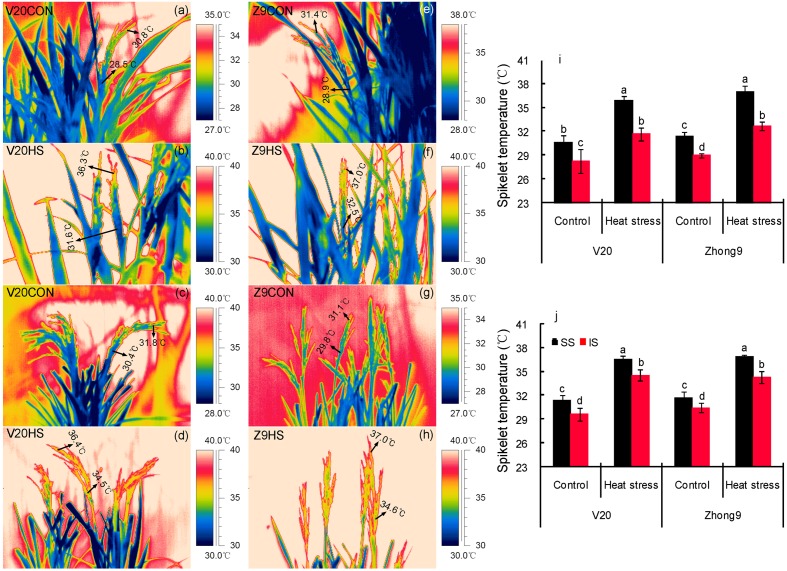
**Thermal images of superior and inferior spikelets with (a–d,i) or without (e–h,j) leaves.** These thermal images were photographed on day 4 after heat stress of 40°C at 10:00 am–11:00 am, avoiding exposing direct sunlight. V20CON and Zhong9CON present genotypes V20 and Zhong9 under control condition, while V20HS and Zhong9HS present V20 and Zhong9 under heat stress. SS and IS were indicated as superior spikelets and inferior spikelets, respectively. The data in the figures **(i,j)** were shown as the mean of 10 replicates. Vertical bars denote standard deviations (*n* = 10). Different letters indicate significant differences between the superior spikelets and inferior spikelets in the same cultivar (*P* < 0.05).

Regardless of natural conditions or heat stress, the temperatures of superior spikelets were always significantly higher than those of inferiors for both rice genotypes (**Figure 4**). Approximately 2.40 and 2.36°C temperature differences were shown between the superior and inferior spikelets of V20 and Zhong9, respectively, under control conditions (**Figures 4a,e,i**), while they increased to 4.22 and 4.28°C, respectively, under stress (**Figures 4b,f,i**). Surprisingly, temperature differences over 7.0°C between the superior and inferior spikelets was also observed in both genotypes (data not shown).

The temperature differences between superior and inferior spikelets were reduced if the flag leaves were removed. Temperature differences between the superior and inferior spikelets in V20 and Zhong9 under control conditions were approximately 1.75 and 1.27°C, respectively (**Figures 4c,g,j**), while they only increased to 2.03 and 2.54°C under heat stress (**Figures 4d,h,j**). Generally, the spikelet temperatures increased after removing the flag leaves, especially for inferior spikelets.

**FIGURE 5:**
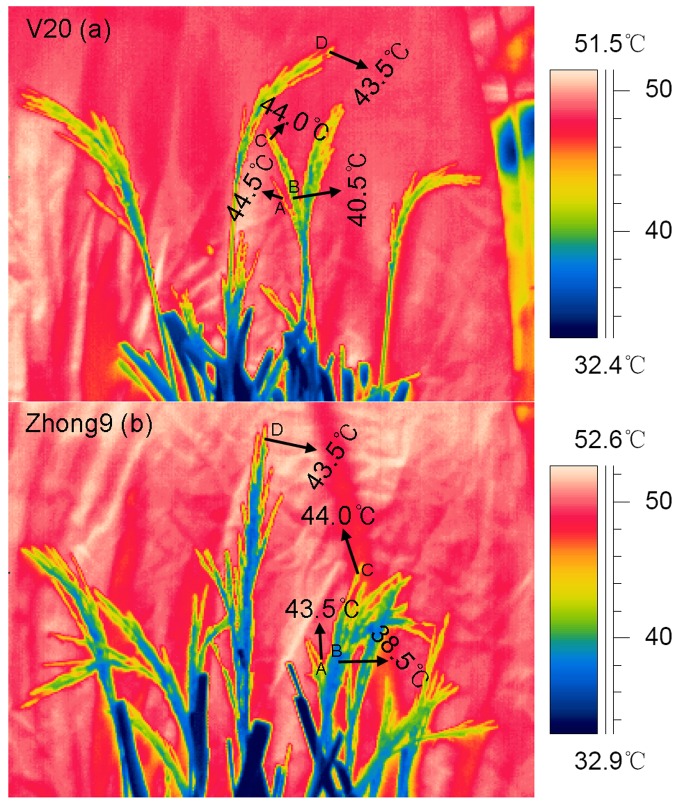
**Thermal images of spikelets on the branches in different located position within a panicle without leaves.** These thermal images were photographed on day 4 after heat stress of 43°C at 10:00 am–11:00 am in another green house under the natural sunlight. In both genotypes, location A, and B were under the same canopy height, while B, C, and D located in different canopy height. **(a)** V20 and **(b)** Zhong9.

Interestingly, the temperature differences existed between the superior and inferior spikelets with or without the flag leaves under heat stress, although the difference in the former was higher than the latter (**Figures 4** and **5**). When the rice plants were subjected to heat stress of 43–44°C (**Figure 5**), obvious temperature differences were shown between those spikelets located in A and B, which were at the same canopy height, in both genotypes. However, no remarkable difference was observed among those spikelets located in A, C, and D with different canopy heights. These results indicated that, in this case, the spikelet temperature was determined by the panicle types rather than the canopy heights or positions.

### Canopy Temperature, Relative humidity, and Illumination intensity

**FIGURE 6:**
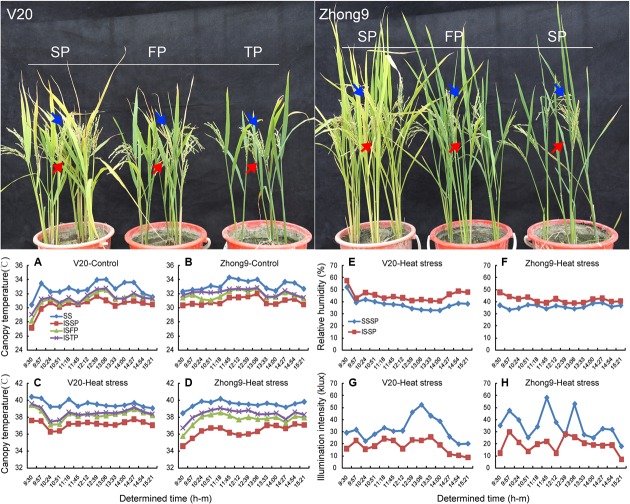
**The air temperature, relative humidity, and illumination intensity determined in the canopy which the superior and inferior spikelets located, respectively.** The arrows showed in blue represent the canopy the superior spikelets (SS) located, and the arrows showed in red represent the canopy the inferior spikelets (IS) located. **(A,C,E,G)** V20, **(B,D,F,H)** Zhong9. **(A,B)** were determined under natural condition, while **(C–H)** were determined under heat stress. SP, FP, and TP present each rice plant possessing six panicles, four panicles, and two panicles, respectively.

The air temperature within the canopy decreased as the canopy height was reduced either under natural conditions or heat stress (data not shown). Under a natural condition with a 33–34°C air temperature, the mean temperatures of the canopy in which the superior spikelets located were approximately 32.7 and 33.2°C, respectively, in the rice genotypes V20 and Zhong9, while those of the inferiors were approximately 30.4 and 30.9°C (**Figures 6A,B**). When the rice plants were subjected to the heat stress of 40 to 41°C, the mean air temperature differences between the canopies where the superior and inferior spikelets located were increased to 3.2 and 2.4°C in V20 and Zhong9, respectively (**Figures 6C,D**). Nevertheless, this difference was reducing as the panicle numbers per plant were decreased. There were only 1.5 and 1.1°C temperature differences shown in those rice plants with FP and TP, respectively (**Figures 6A–D**). Additionally, the illumination intensity above the canopy was also obviously higher than that below under heat stress (**Figures 6E,F**). In contrast, lower relative humidity was shown in the former than the latter (**Figures 6G,H**).

### Transpiration Rate and Relative Water Content

**FIGURE 7:**
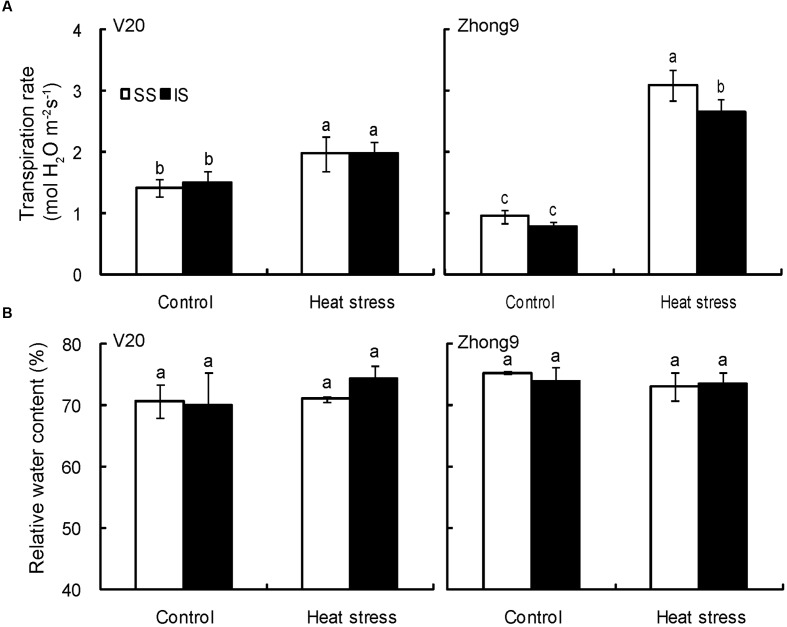
**Effect of heat stress on the transpiration rate (A) and relative water content (B) of superior and inferior spikelets at anthesis of rice.** SS and IS were indicated as superior spikelets and inferior spikelets, respectively. The data in the figures **(A,B)** were showed as the mean of 10 replicates. Vertical bars denote standard deviations (*n* = 3). Different letters indicate significant differences between the superior spikelets and inferior spikelets in the same cultivar (*P* < 0.05).

The spikelet transpiration rates were significantly increased in both genotypes under heat stress compared to their respective control, particularly in Zhong9 (**Figure 7A**). Nevertheless, little difference in transpiration rates was observed between the superior and inferior spikelets whether under natural condition or heat stress except for the Zhong9 under heat stress (**Figure 7B**). The transpiration rate of the superior spikelets was obviously higher than that of inferiors of Zhong9 under stress condition. Surprisingly, the RWC of the spikelets was not determined by their positions or temperature settings because no difference was shown between the superior and inferior spikelets either under control condition or heat stresses in both genotypes (**Figure 7B**).

### Pollen Viability, Pollen Numbers and Pollen Germination on the Stigma, and Stigma ROS

**FIGURE 8:**
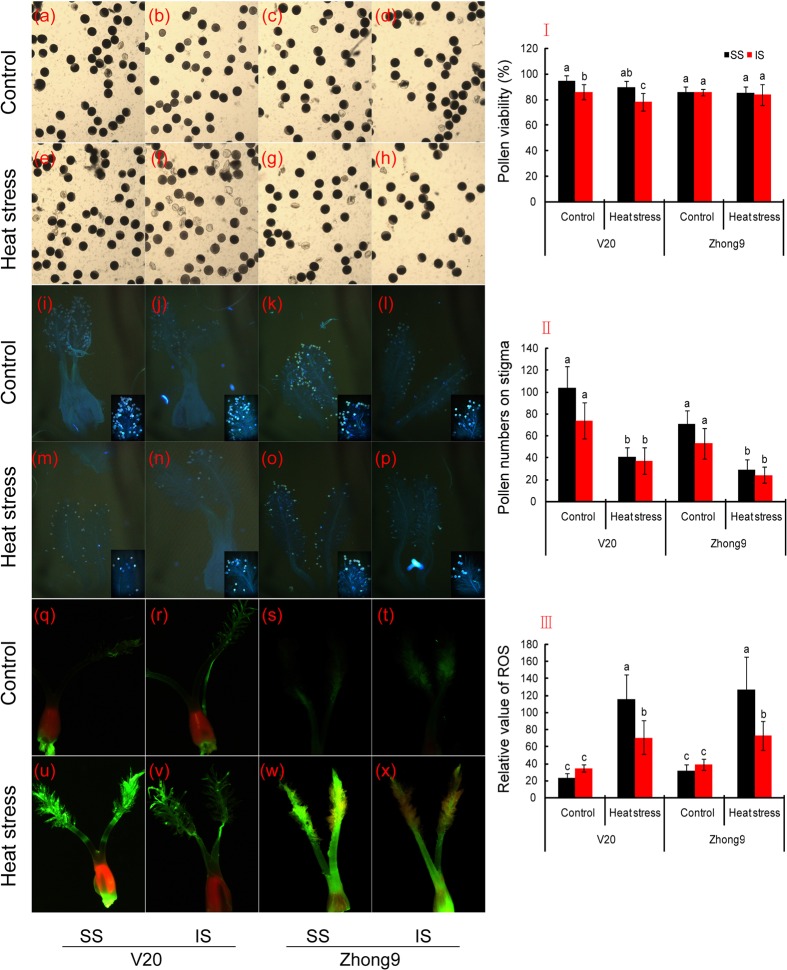
**Changes in the pollen viability, pollen number and pollen germinating on stigma, and pistil ROS between superior and inferior spikelets.** SS and IS were indicated as superior spikelets and inferior spikelets, respectively. **(a–d,I)** were pollen viability, **(i–p,II)** was pollen numbers and pollen germinating on stigma, and **(q–x,III)** were pistil ROS. The data in the figures **(I,II,III)** were showed as the mean of 10 replicates. Vertical bars denote standard deviations (*n* = 10). Different letters indicate significant differences between the superior spikelets and inferior spikelets in the same cultivar (*P* < 0.05).

For the genotype V20, higher pollen viability was observed in superior spikelets than inferiors under both natural condition and heat stress, in particular the latter (**Figures 8a,b,e,f,I**). Heat stress caused little effect on the pollen viability of superior spikelets, but significantly decreased that of inferior spikelets of V20. The pollen viability of Zhong 9 was not affected by heat stress that no obvious difference showed between the heat stress treatment and normal temperature treatment (**Figures 8c,d,g,h,I**). Furthermore, there was no significant difference in pollen viability shown between the superior and inferior spikelets under either natural condition or heat stress.

Heat stress obviously decreased the pollen number on the stigma of superior and inferior spikelets in both rice genotypes, especially in the superior spikelets. We found that a remarkable decrease was shown in superior spikelets compared to inferiors under heat stress (**Figures 8i–p,II**). Surprisingly, pollen germination was found on the stigma of superior and inferior spikelets under both natural and stress conditions.

In terms of stigma ROS values, no obvious difference was observed between the superior and inferior spikelets under natural conditions in both rice genotypes (**Figures 8q–x,III**). Nevertheless, ROS were induced by heat stress and were significantly increased in both superior and inferior spikelets compared to their respective controls. In sum, the relative value of ROS in superior spikelets was obviously higher than that of inferior spikelets under heat stress.

### Starch Branching Enzyme Activity and Soluble Sugar Content

**FIGURE 9:**
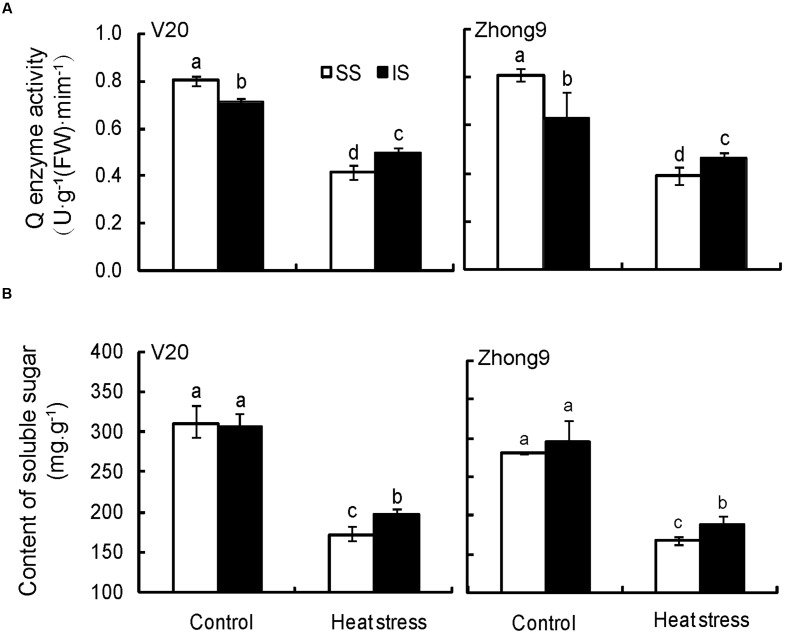
**Effect of heat stress on the starch branching enzyme (Q enzyme) activity (A) and soluble sugar content (B) of superior and inferior spikelets at anthesis of rice.** SS and IS were indicated as superior spikelets and inferior spikelets, respectively. Vertical bars denote standard deviations (*n* = 4). Different letters indicate significant differences in the same cultivar (*P* < 0.05).

Heat stress at anthesis significantly decreased the starch branching enzyme (Q enzyme) activity of superior and inferior spikelets in both genotypes, especially in the former (**Figure 9A**). Under natural condition, higher starch branching enzyme activity was observed in superior spikelets than inferiors, whereas that of the former was significantly lower than the latter when encountering heat stress in both genotypes.

There was no obvious difference in soluble sugar between the superior and inferior spikelets in both genotypes under natural condition (**Figure 9B**). When rice plants were subjected to heat stress, the soluble sugar of inferior spikelets was obviously higher than that of the superior spikelets.

### Phytohormones

The phytohormones, including ZR, IAA, ABA, and GA3, were determined on day 14 of the heat stress treatment. Heat stress caused little effect on ZR, and there was no obvious difference between superior and inferior spikelets under either natural condition or heat stress (**Table 1**). Under natural condition, the IAA concentration of superior spikelets was 66.1 and 39.5% higher than those of inferiors in V20 and Zhong9, respectively, while the difference was reduced under heat stress. Only a 14.7% higher concentration of IAA was found in superior spikelets than inferior spikelets in Zhong9 under heat stress, while it changed to a higher IAA concentration in the inferior spikelets than superiors in V20. Heat stress significantly decreased the ABA concentration at anthesis, in which a higher decrease was shown in the inferior spikelets of V20, whereas for Zhong9 it was shown in the superior spikelets. The GA_3_ concentration of spikelets in V20 was not affected by heat stress, and no obvious difference was demonstrated between the superior and inferior spikelets. Heat stress significantly decreased the GA_3_ concentration of superior spikelets in Zhong9, whereas a great increase was shown in the inferior spikelets under heat stress compared with that under natural condition.

**Table 1:** Effect of heat stress on the phytohormones of superior and inferior spikelets of rice at anthesis.

Genotypes	Treatments	Spikelets Position	ZR (ng⋅g^–1^⋅FW)	IAA (ng⋅g^–1^⋅FW)	ABA (ng⋅g^–1^⋅FW)	GA_3_ (ng⋅g^–1^⋅FW)
V20	Control	Superior	5.72 ± 1.57a	87.67 ± 3.98a	84.95 ± 4.57a	7.71 ± 0.26a
		Inferior	6.18 ± 0.49a	52.79 ± 9.13b	93.51 ± 6.00a	6.78 ± 0.82a
	Heat stress	Superior	6.13 ± 0.20a	17.07 ± 2.95c	64.52 ± 6.81b	7.86 ± 0.19a
		Inferior	7.18 ± 1.88a	45.23 ± 2.12b	64.62 ± 7.16b	7.15 ± 0.90a
Zhong9	Control	Superior	6.09 ± 1.21a	61.68 ± 5.03a	93.80 ± 3.58a	8.03 ± 0.51ab
		Inferior	4.74 ± 0.63a	44.20 ± 1.93b	95.98 ± 2.16a	5.65 ± 0.88c
	Heat stress	Superior	4.85 ± 0.62a	40.32 ± 1.97bc	63.43 ± 2.75b	6.70 ± 0.58bc
		Inferior	5.86 ± 1.07a	34.49 ± 2.96c	89.82 ± 0.78a	9.43 ± 1.64a

*ZR, zeatin riboside; IAA, indole acetic acid; ABA, abscisic acid; GA_*3*_, gibberellins; FW, fresh weight. Different letters behind the data indicate statistically significant differences between the superior and inferior spikelets in the same cultivar (P < 0.05).*

## Discussion

### Factors Involved in Temperature Differences between Superior and Inferior Spikelets of Rice

In the present study, a higher decrease in fertility and kernel weight were observed in superior spikelets than inferiors under heat stress (**Figures 1** and **2**). This suggested that heat stress at anthesis was more damaging to the superior spikelets than the inferior spikelets. Obviously, this finding was different from those previous results that abiotic stress, including drought, heat and low temperature stress, caused more injury to the inferior spikelets than the superiors (Yuan et al., 2006; Dong et al., 2011; Zhang et al., 2012; Yang D.Q. et al., 2014; Zhao et al., 2014). It is well-known that the competition for assimilation exits between the superior and inferior spikelets (Stansel, 1975; Wang et al., 2001), and this completion could be more exacerbated when encountered drought (Chen et al., 2013) and high night temperatures stress (Mohammed and Tarpley, 2010). Thus, we inferred that the temperature differences mainly led to the different responses of superior and inferior spikelets to heat stress because the temperatures of superior spikelets were significantly higher than those of inferior spikelets (**Figure 4**). The temperatures of superior spikelet were 2.40 and 2.36°C higher than those of inferior spikelet in V20 and Zhong9 under natural condition, respectively, while the temperature difference were increased to 4.22 and 4.28°C under heat stress (**Figure 4**). This means that when the temperatures of superior spikelets reached 36.0°C, the inferior spikelet temperature might be less than 33°C. In this case, there is no doubt that superior spikelets might suffer more stress damage than the inferior spikelets.

Many factors, such as the organ size, shape, location within the canopy and cooling ability as well as the environment, i.e., the air temperature, relative humidity, wind speed and radiation, are involved in mediating the spikelet temperature (Sheehy et al., 1998; Yan et al., 2008, 2010; Zhang et al., 2016). These factors can lead to different surface temperatures for identical objects in the same air temperature (Zhang et al., 2016). It has been reported that rice panicles can attain temperatures that are more than 6°C lower than the ambient temperature when atmospheric evaporative demand is high, whereas the panicle temperature can exceed the air temperature by 4°C under humid conditions (Matsui et al., 2007; Tian et al., 2010). Among these factors, the spikelet location might play a main role in the temperature differences between the superior and inferior spikelets. Within one panicle, the superior spikelets were almost 20–25 cm higher than the inferior spikelets of both rice genotypes. Accordingly, the air temperature of the canopy in which the superior spikelets located was approximately 2.0–3.0°C higher than that of the inferior spikelets (**Figures 6A–D**). The results agreed with Ayeneh et al. (2002) and Yan et al. (2008), who reported that the temperatures of the second or third leaves were lower than those of the flag leaves within the same rice plants, and plant organs (leaf, peduncle, and spike) may also have different temperatures as a result of their position within the canopy. Importantly, the superior spikelets received more illumination than the inferiors due to their different positions within the canopy, possibly because of the shade effect by the leaves (**Figures 6G,H**), which can exacerbate the temperature differences between the superior and inferior spikelets. We observed that the spikelet temperature could obviously increase by approximately 3–4°C when exposed to direct sunlight (data not shown). This finding was supported by the results of Pararajasingham and Hunt (1991), who had reported spikelet temperatures of wheat grown outdoors with an adequate water supply was 1.5°C greater than air temperature, while the spikelet temperature was found to be 3–4°C above that of air when the lights were on.

Whether with or without flag leaves, temperature differences existed between the superior and inferior spikelets (**Figure 4**). Because there was no obvious difference in the transpiration rates between the superior and inferior spikelets under both natural and stress conditions (**Figure 7A**), which played an important role in reducing the plant organ temperatures (Zhang et al., 2016), we inferred the panicle type might be another important factor contributing to this temperature difference. As shown in **Figure 5**, higher spikelet temperatures were observed in location A than B, which had the same canopy height, while no obvious difference was shown among locations B, C, and D at different canopy heights, indicating the panicle type, rather than the canopy height, mainly contributed to the temperature differences without leaves. This was confirmed by the results of Yan et al. (2010), who reported that lower organ temperatures were recorded in the rice cultivars with erect panicles than those with droopy panicles under similar climatic conditions. Based on our observations, the erect panicle always belonged to compact panicles, while a drooping panicle belonged to loose panicles. This property could result in higher contact areas to air in the erect panicle than a droopy panicle. In this case, the former might absorb more heat energy than the latter, leading to higher organ temperatures in droopy panicles. For most rice cultivars, the three apical primary branches the superior spikelets were located on were always loose, while those the inferior spikelets were located on were compacted. This finding could explain the higher temperatures shown in the superior spikelets than the inferior spikelets when the leaves were removed.

### Mechanism of Heat Damage to Spikelet Fertility and Kernel Weight

There was no doubt that the organ temperatures were obviously related to the degree of damage induced by heat stress, and thus higher decreases in fertility and kernel weights shown in the superior spikelets than inferiors was mainly due to their different organ temperatures. Heat stress at anthesis inducing spikelet sterility was mainly ascribed to inhibiting anther dehiscence (Matsui and Omasa, 2002; Matsui et al., 2005), pollen germination (Song et al., 2001; Wassmann and Dobermann, 2007) and pollen tube elongation (Tang et al., 2008). Our results confirmed that heat stress significantly decreased the pollen numbers on the stigma, especially the superior spikelets (**Figures 8i–p,II**). Interestingly, this stress caused little effect on the pollen viability and pollen germination on the stigma, which was obviously different from previous results that required further study. Thus, stigma ROS might be the main factor responsible for the higher sterility in the superior spikelets than inferiors, possibly because higher ROS were shown in the pistils of superior spikelets (**Figures 8q–x,III**), which could disturb the pollen tube entering into the ovule. In addition, the flowering time might be another important factor. It is well-known that the superior spikelets flower earlier, thus suffering heat stress first. These sterile spikelet increases would benefit those spikelets that flowered later, including the inferior spikelets, because the competition for assimilation exists between the superior and inferior spikelets (Stansel, 1975; Wang et al., 2001). This effect was similar to removing the superior spikelets to reduce apical-grain superiority, and the inferior spikelets would attain more assimilation, which was important for these spikelets to survive in heat stress.

The greater decrease in kernel weight of superior spikelets than inferiors caused by heat stress might mainly ascribe to the changes in the activity of Q enzymes and IAA concentration, which had been reported to play an important role in mediating the grain-filling in spikelets (Yang et al., 2002; Tao et al., 2003; Abu-Zaitoon et al., 2012). We observed that heat stress significantly decreased the Q enzyme and IAA in the spikelets in both genotypes, but higher reduce were shown in superior spikelet than inferiors. This changing pattern was similar to the temperature difference between the superior and inferior spikelets under heat stress, suggesting that the key enzymes including Q enzyme and IAA in spikelets were susceptible to heat stress, which had been confirmed by the previous results (Jin et al., 2005; Tang et al., 2008; Ahmed et al., 2015). Additionally, the greater reduction in the content of IAA shown in superior spikelets induced by heat stress could reduce the apical-grain superiority to mediate grain-filling in inferior spikelets (Wang et al., 2001, 2003).

### Relationship between Canopy Temperatures and Plant Architecture of Rice

We observed that the canopy temperatures increased when reducing the panicle numbers per pot, confirming an obvious relationship existed between the canopy temperatures and the plant architecture (**Figure 6**). Accordingly, the spikelet fertility and kernel weight decreased as the canopy temperature increased under heat stress, especially the inferior spikelets (**Figure 2**). These findings provide a reference for the ideotype breeding of rice with heat resistance, which has been considered to be effective for breaking the yield ceiling of the irrigated rice crop (Peng et al., 2008). The rice ideotype consists of those morphological and physiological traits that will contribute to higher yields than the currently prevalent crop cultivars, and this approach has been used in breeding programs in the International Rice Research Institute (IRRI) in Philippines and China to improve the rice yield potential. However, Jin et al. (2013) suggested that in ideotype breeding practice, the criteria should be regionalized based on the local climatic and cultivation conditions, which should consider not only the traits of “space” but also the specific geoecotypes. Because certain plant-types of rice are suitable to grow in southern China (Zhou, 1995; Huang, 2001), while some are widely planted in northern China (Yang et al., 1996), the differences are partly ascribed to their different morphological traits and ecological environments including the humidity, temperature and radiation. These results mean that some of the morphological traits might not be suitable for rice-growing areas with high air temperatures. For example, the characteristic features including upright growth habits, and fewer, well-spaced, thick, large but stiff leaves able to maintain an erect position, can increase the light transmission rate within the canopy, which is favorable for photosynthesis. Nevertheless, this feature can also obviously increase the canopy temperature due to the higher light transmission rate (Yan et al., 2008). Furthermore, the plant organ temperatures with light were significantly higher than those without light under the same condition. Thus, we inferred that the plant feature with an upright growth habit, and fewer, well-spaced, thick, large but stiff leaves able to maintain an erect position might cause more damage to rice plants under heat stress. This was mainly ascribed to their higher canopy temperatures, which was suggested to be one of the important criteria for the selection of stable genotypes under heat and drought stress (Saint Pierre et al., 2010; Gautam et al., 2015).

## Conclusion

Heat stress occurring at anthesis caused more damage to the superior spikelets than the inferiors of rice, which was mainly ascribed to their different organ temperatures. Three factors, including canopy temperature, illumination intensity, and plant type, were involved in mediating the organ temperatures of superior and inferior spikelets. The temperature and illumination intensity within the canopy where the superior spikelets located were significantly higher than that where the inferior spikelets were located. Additionally, the three apical primary branches the superior spikelets located on were always loose, while the three base primary branches the inferior spikelets located on were compacted. Accordingly, the contact area of the superior spikelets to air was much greater than those of inferior spikelets, suggesting the former absorbed more heat energy than the latter. Heat stress occurring at anthesis significantly increased spikelet sterility, and higher reduce were shown in superior spikelet than inferior, which was mainly ascribed to the remarkable decrease in pollen numbers on the stigma, as well as an increase in ROS, especially for the superior spikelets. The obvious decrease in kernel weight of superior spikelets induced by heat stress resulted from their lower Q enzyme activity and IAA content. Additionally, the spikelet fertility and kernel weight decreased as the panicle numbers per plant were reduced under stress, especially for inferior spikelets, which was mainly due to their higher canopy temperature. This finding suggested that those rice plants with characteristic features of upright growth habits and maintenance of an erect position were more susceptible to heat stress due to their higher canopy temperatures.

## Author Contributions

Conceived and designed the experiments: GF, QJ, and LT. Performed the experiments: GF, BF, CZ, and XiaZ. Analyzed the data: YY and TC. Contributed reagents/materials/analysis tools: XY, XiuZ, and GF. Wrote the paper: GF and BF.

## Conflict of Interest Statement

The authors declare that the research was conducted in the absence of any commercial or financial relationships that could be construed as a potential conflict of interest.
